# Heart Disease

**Published:** 1859-04

**Authors:** 


### HEART DISEASE.

When an individual is reported to have died of a "Disease of the Heart," we are in the habit of regarding it as an inevitable event, as something which conld not have been foreseen or prevented, and it is too much the habit, when persons suddenly fall down dead, to report the "heart" as the cause; this silences all inquiry and investigation, and saves the trouble and inconvenience of a repulsive "post mortem." A truer report would have a tendency to save many lives. It is through a report of "disease of the heart," that many an opium eater is let off into the grave, which covers at once his folly and his crime; the brandy drinker too, quietly slides round the corner thus, and is heard of no more; in short this "report" of "disease of the heart," is the mantle of charity, which the politic coroner, and the sympathetic physician throw around the grave of "genteel people."

At a late scientific congress at Strasburgh, it was reported, that of sixty-six persons who had suddenly died, an immediate and faithful post mortem showed that only two persons had any heart affection whatever: one sudden death only, in thirty three, from disease of the heart. Nine out of the sixty-six died of apoplexy, one out of every seven, while forty-six, more than two out of three, died of lung affections, half of them of "congestion of the lungs," that is, the lungs were so full of blood, they could not work, there was not room for air enough to get in to support life.

It is then of considerable practical interest to know some of the common every day causes of this "congestion of the lungs," a disease which, the figures above being true, kills three times as many persons at short warning, as apoplexy and heart disease together. Cold feet; tight shoes; tight clothing; costive bowels; sitting still until chilled through and through after having been warmed up by labor or a long or hasty walk; going too suddenly from a close heated room, as a lounger or listener or speaker, while the body is weakened by continued application, or abstinence, or heated by the effort of a long address; these are the fruitful, the very fruitful causes of sudden death in the form of "congestion of the lungs;" but which being falsely reported as "disease of the heart," and regarded as an inevitable event, throws people off their guard, instead of pointing them plainly to the true causes, all of which are avoidable, and very easily so, as a general rule, when the mind has been once intelligently drawn to the subject.

